# Physical activity and neurocognitive functioning in aging - a condensed updated review

**DOI:** 10.1186/s11556-016-0161-3

**Published:** 2016-01-21

**Authors:** Patrick D. Gajewski, Michael Falkenstein

**Affiliations:** Leibniz Research Centre for Working Environment and Human Factors (IfADo), Ardeystr. 67, D-44139 Dortmund, Germany; Institute for Working, Learning and Aging, Bochum, Germany

**Keywords:** Aging, Aerobic exercise training, Physical activity, Executive functions, ERP, fMRI

## Abstract

This condensed review gives an overview about two methodological approaches to study the impact of physical activity on cognition in elderly, namely cross-sectional studies and randomized controlled intervention studies with pre- and post-measures. Moreover, this review includes studies investigating different types of physical activity and their relation to cognitive functions in older age. Behavioral data are considered but the main focus lies on neuroscientific methods like event-related potentials (ERPs) and functional magnetic resonance imaging (fMRI).

## Background

Healthy aging is associated with a decline in sensory, motor and specific cognitive functions. However, such declines depend on various factors, such as genetics and lifestyle [1]. In particular, physical activity and training not only improve physical and motor but also cognitive functions [2–11] and reduce risk for cognitive decline and dementia in later life [8, 12–19]. Generally, executive functions that control lower-level functions appear to benefit most from physical activity as documented in several review articles [2, 20, 21]. Neurophysiological intervention studies show both general effects such as an increased cerebral blood flow and specific effects on certain brain structures and functions [5, 11, 22]. Different types of exercise appear to exert distinct effects on brain and cognition [23].

Cross-sectional studies overall show a positive association between regular physical exercise and cognitive functions. However, in this type of studies the causal relationship between physical activity and cognitive functions cannot be unequivocally drawn. Nevertheless, meta-analyses of behavioral and neuroscientific studies showed stable associations between physical fitness and cognitive, mainly executive functions in older age [20]. Randomized intervention studies with control groups that represent a more valid method to draw causal relationship between variables even show more consistent effects on cognition in older adults [2, 5, 24], but see [25].

The aim of the next two sections is providing an overview about cognitive changes associated with physical activity in elderly individuals across electrophysiological and fMRI studies using cross-sectional and randomized-controlled trial designs.

## Cross-sectional (correlative) studies

Structural MRI studies revealed a consistent positive relationship between cardiorespiratory fitness and both brain volume and functional activation in cortical regions including anterior cingulate, lateral prefrontal, and lateral parietal cortex [5, 11]. Also higher physical fitness is associated with greater gray matter volume in the hippocampus [26]. Those regions are linked to cognitive, in particular executive and memory functions, which are known to deteriorate with increasing age. Indeed, high physical activity is related to high cognitive performance [8]. The review of Lautenschlager et al. [27] also suggests a robust and dose-dependent relationship between physical exercise and cognitive performance in older adults. Concerning the specificity of cognitive functions Desjardins-Crépeau et al. [28] accumulated evidence that high physical fitness was associated with greater processing speed and better executive functions while memory performance assessed by the Ray Verbal Memory Test (RAVLT) was less improved. The fMRI study conducted by Prakash and co-authors [22] found less interference and higher accuracy accompanied by enhanced activity in anterior brain regions in physically active seniors in several executive control tasks. Another correlational study using a larger sample size of older adults yielded association between older adults’ physical fitness and their reaction times on the incompatible trials of the flanker task, such that physically active adults responded faster and showed higher working memory capacity during 2-back task than their low active peers [29]. In a longitudinal correlational study of about 1400 participants aged 19–94 years, Wendell et al. [30] found that neuropsychological performance was positively associated with maximum oxygen consumption. In a longitudinal correlational study with nearly 5000 participants, Chang et al. [31] found a significant association between midlife habitual physical activity and executive functions measured 25 years later. These data suggest that long-term physical activity may counteract age-related decline of executive functions. Generally, previous research indicates that older adults who engage in physically active recreational activities or have higher cardiovascular fitness are at lower risk for cognitive decline compared to inactive older adults [12–19, 21, 22, 25, 32–37].

Event-related potentials derived from the electroencephalogram (EEG) during performance of cognitive tasks offer insights in the mechanisms underlying sensory and cognitive functions. As the ERPs have excellent time resolution, there is the possibility to analyze each process separately. Different ERP components reflect specific sensory, cognitive and central motor functions and can thus help pinpointing the origin of behavioral effects. However, due to a low spatial resolution of this method the spatial aspect is less informative. Instead, more informative are temporal and functional properties of the neurobehavioral gains due to physical activity. It has been shown that not only performance in executive tasks and fMRI activity pattern but also some ERP components are modulated by physical activity [9, 20]. Using a cross-sectional design, Berchicci et al. [38] investigated movement-related cortical potentials (MRCPs) in a sample of 130 participants between 16 and 86 years of age, divided into regularly physically active and low active groups. They found faster responses and shorter latency of MRCPs in the former than the latter group. Interestingly, the magnitude of speed and latency differences began to increase after age of 30, suggesting that older people benefit more from physical activity than younger ones. Other cross-sectional studies also observed a positive relationship between physical activity and cognitive functions in elderly. For example, Taddei and colleagues [39] used a cross-sectional design to study performance of young fencers, older fencers, and non-fencers in a go/no-go task. In keeping with previous research on young fencers [40], they reported faster reaction times and an earlier and larger N2 in fencers compared to non-fencers. Hillman et al. [41] found lower mixing and switching costs and shorter latencies and larger amplitudes of the P3b component in physically active than inactive older individuals. Themanson et al. [42] reported lower mixing costs but not switching costs in active vs. low active older adults. More recently, Dai and colleagues [43] compared three groups of older adults with open-skills (e.g. tennis), close-skills (e.g. jogging) and irregular physical activity. The total duration of the physical activity between groups varied between 11 and 13 years. The open skill groups revealed the lowest mixing costs, following by the closed-skill and the no activity groups. No effects on local switching costs were obtained. The ERPs showed larger P3b amplitudes in both active groups supporting the findings of Hillman et al. [41]. In a recent cross-sectional study Gajewski and Falkenstein [44, 45] investigated the impact of life-long physical activity (about 50 years) on executive functions and ERPs. The authors compared two groups of either physically high or low active healthy older men. Physical activity was quantified in three different time scales: long-term activity (across decades) was assessed by self-reports, mid-term (last 2 years) activity was assessed by a questionnaire with detailed information about the weekly time spending on physical activity and current activity was assessed by a bicycle ergometry. The groups differed in all three dimensions significantly. In other respects the groups were carefully matched. The study targeted executive functions, namely inhibition (Stroop task), task switching and working memory (memory based switch task). In both tasks physically active seniors showed better behavioral performance, particularly under interference and task switching needs. The interference score in the Stroop task was negatively correlated with physical activity. In both tasks the ERPs revealed a shorter latency of the P2, reflecting faster recall of stimulus-response mappings and generally more negative amplitudes over fronto-central brain regions like N2 and N450, indicating enhanced inhibitory processing in the high active than the low active group. Similar enhanced frontocentral activity was observed in young vs. old subjects (unpublished data), suggesting that the ERP pattern (e.g. amplitudes, timing or even morphology of ERP components) in physically active seniors becomes similar to the ERPs observed in young adults. A further study tested auditory distraction while the subjects had to respond to short and long auditory stimuli [46]. Occasionally the auditory stimuli had a slightly different frequency which was task-irrelevant. The frequency deviations impaired performance more in physically low active than high active seniors. This was accompanied by a stronger frontal positivity (P3a) and increased activation of anterior cingulate cortex, suggesting a stronger involuntary shift of attention towards task-irrelevant stimulus features in low active compared to highly active seniors. The results showed also a positive relationship between physical fitness and attentional control, presumably due to more focused attentional resources and enhanced inhibition of irrelevant stimulus features in physically active seniors.

As in the review of Desjardins-Crépeau [28] in the reported study the short- and long-term memory performance did not differ between physically high active and low active individuals. Also other tasks such as visual search and simple Go/Nogo tasks showed no group difference. Hence, it appears that not all but specific executive functions (mostly inhibition of distracting events) are improved in older subjects by lifelong physical activity.

Finally, a recent review by Prakash et al. [21] provides evidence for physical activity to be associated with a modest reduction in relative risk of cognitive decline. An evaluation of the physical activity-cognition link across the life span provides modest support for the effect of physical activity on preserving and even enhancing cognitive vitality and the associated neural circuitry in older adults, with the majority of benefits seen for tasks that are supported by the prefrontal cortex and the hippocampus.

## Randomized controlled intervention studies with pre- and post-measure

As shown, most of the cross-sectional studies reveal good evidence for the positive association between physical exercise and cognition, particularly executive functions in older population. However, given the correlative nature of these studies, causation cannot be established. Therefore, a number of randomized, controlled intervention studies with pre- and post- measures have been conducted with older adults. The duration of such trainings was very different. Berryman et al. [47] compared the effects of different short-term (8 week) physical interventions in 51 older adults. All groups showed similar improvements in cognition, with maximum effect on inhibition. Forte et al. [48] trained 42 older adults for 3 months in either coordination (e.g. multicomponent training, prioritizing neuromuscular coordination, balance, agility, and cognitive executive control) or classic resistance training for muscle strength conditioning like machine exercises. Inhibitory control improved after the intervention, independent of training type. Similarly, Liu-Ambrose et al. [49] found an improved inhibitory control using a Stroop test after 12 months resistance training relative to the control group. Predovan et al. [50] reported lower interference susceptibility in seniors after 3 months aerobic training study relative to non-trained persons. Increase in physical capacity was associated with lower interference scores. Albinet et al. [51] trained older adults with an aerobic exercise vs. a stretching program for 12 weeks. Executive control was measured with the Wisconsin Card Sorting Test. Only the participants in the aerobic group improved their test performance. These results confirm that physical training improves executive functions. However, also some specific memory functions appear to profit from physical training. Erickson et al. [52] administered a 12 months aerobic exercise program to sedentary older adults. The active participants showed an improvement in spatial memory.

Colcombe and Kramer [2] conducted a meta-analysis of randomized controlled trials concerning the effect of physical training on cognition in healthy older adults. They obtained a stable effect of physical training with moderate effect size (0.48). The largest effects size was observed for executive control processes (0.68). The effects were larger when aerobic training was combined with strength and flexibility training. In addition, session duration of at least 30 min and the total duration of at least 6 months appear to be necessary to produce stable effects on cognition. A later review of Kramer and Erickson [8] suggests a moderate-intensity exercise on average one hour per session and frequency at least 3 sessions per week show greater cognitive and brain effects.

A more recent meta-analysis including randomized controlled studies from 1966 until 2009 with a total number of 2,049 participants showed modest improvements in processing speed, attention and executive functions, whereas memory effects were less consistent [24]. A meta-analysis by Hindin and Zelinski [53] indicates that aerobic exercise interventions have moderate to medium-sized effects on executive function and memory. The review of Kirk-Sanchez and McGough [54] stated that many studies demonstrate positive effects of exercise on cognitive performance, while others show minimal to no effect. This is line with the recent meta-analysis of Kelly et al. [25] that analyzed 25 randomized controlled studies, investigating cognitive effects of aerobic exercise, resistance training and Tai Chi. The authors found evidence that resistance training and Tai Chi have cognitive benefits among seniors, whereas no consistent significant effects were found for aerobic exercise. Hence, the variables that influence the impact of physical activity on cognition have to be explored in more detail in the future. Important variables are training type, frequency, duration and intensity but also factors like age, base level of physical performance and sensory and general cognitive abilities have to be considered.

Several recent studies investigated different types of physical training. Voelcker-Rehage et al. [23] compared a 12-month cardiovascular with coordination training in older adults. Changes in brain activation were investigated by functional magnetic resonance imaging (fMRI). Both groups improved in executive functioning and perceptual speed. The fMRI revealed unspecific changes for both groups in prefrontal areas, and also training-specific changes in other brain regions. In a follow-up study the group showed that motor fitness due to coordination training lead to an increase of subcortical brain areas relevant for motor control [55]. Recent reviews have summarized the relationship between physical activity and cognitive functions in elderly and focused on different types of physical activity like aerobic, resistance and coordination training and evaluated the underlying neuronal mechanisms [21, 56, 57].

Physical training interventions result in various effects on brain structure and function. Gomez-Pinilla and Hillman [9] proposed that exercise influences cognition by affecting molecular events related to the management of energy metabolism and synaptic plasticity. Chapman et al. [57] found increased cerebral blood flow (CBF) and a related increase of memory performance in older adults after three months of physical training. In the 12-months-study of Erickson [52] cited above the participants who completed a 12-month aerobic training program showed an increase of the volume of the anterior hippocampus, which was related to an improvement in spatial memory. Increased hippocampal volume was associated with greater serum levels of BDNF, a mediator of neurogenesis. Different types of physical activity affect specific neurotrophins relevant for neural plasticity. Whereas aerobic exercise up-regulates metabolism of the BDNF, resistance exercise seems to stimulate immune-globulin factor 1 production. Both are thought to facilitate neurogenesis, synaptogenesis and angiogenesis through partly interacting pathways [56]. Thus, combined physical interventions may have the largest impact on neural plasticity.

In a recent study Kleemeyer et al. [58] investigated the effects of a six-months fitness training on hippocampal microstructure and volume in sedentary elderly adults. More positive changes in fitness were associated with more positive changes in tissue density, and more positive changes in tissue density were associated with more positive changes in volume. The authors conclude that fitness-related changes in hippocampal volume may be brought about by changes in tissue density.

In a randomized intervention study Gajewski et al. [59, 60] compared the effects of a combined strength and aerobic training to those of two other active groups (PC-based cognitive training, relaxation training) and a no contact group on 142 healthy seniors (65 and above). The active trainings were administered by skilled trainers for 4 months twice a week with 90 min session length. A battery of psychometric tests was administered before and after the training. While the effects of the cognitive training were largest, also the physical training yielded specific effects on cognition, e.g. on processing speed, executive functions (speed in the interference tables in the Stroop test and PC-based task switching), and some aspects of memory (delayed recall in the VLMT). However, working memory, as measured with the ratio of detected targets in the 2-back task, was not improved. Instead, the reaction times of target detection were faster. Thus, the improvements mainly affected the speed of performance, while quality of performance was less improved, inversely to the effects of cognitive training, which mainly improved accuracy. Hence, physical training may improve performance of elderly in everyday situations where speed rather than accuracy is relevant, such as braking in traffic situations, suggesting a faster coupling of perception with action or alternatively a lower motor threshold without changing perceptual and cognitive abilities. However, no effects on electrophysiological measures, which were used in some of the tests, were found. This may be due to the fact that the training duration and/or intensity were too low to observe stable effects on electrophysiological level.

As with all interventions which require effort and extra time, motivation has to be maximized to yield high compliance of older trainees. Hence, virtual-reality enhanced types of exercise (“exergaming”) may increase motivation and also success. Anderson-Hanley et al. [61] investigated the effect of stationary cycling with virtual reality tours (“cybercycling”) on cognitive functions. The authors found that cybercycling improved executive functions and enhanced BDNF more than traditional exercise. Chao et al. [62] reviewed studies that investigated the effects of the Nintendo Wii™ exergames on cognition, physical function, and psychosocial outcomes in older adults. Indeed, positive effects on physical function, cognition and quality of life were found.

A similar but more everyday-like approach is natural physical activity which consists not only of aerobic and strength but also of coordinative and cognitive exercise. Such a multilevel exercise is dancing. Effects of dancing on cognitive performance as well as on brain structure and functions have been found [63, 64]. Kattenstroth and colleagues [63] investigated effects of a 6-month dance class (1 h/week) in physically non-active elderly men and women. The participants learned step sequences of increasing complexity without a dance partner. Beneficial effects were found for dance-related parameters such as posture and reaction times, but also for cognitive, tactile, motor performance, and subjective well-being. However, the data basis concerning dancing and similar activities is by now very scarce [65]. As a most important everyday life outcome, dancing appears to prevent falls, a major source of illness and death in aged people [66]. Since dancing is much more motivating and joyful as a standard exercise, more intervention studies on different types and formats of dancing are necessary. Such studies should also use neurophysiological measures in order to shed more light on the effects of such interventions at the brain level. In particular the electroencephalogram and event-related potentials should be used more frequently to unveil changes of brain mechanisms due to the interventions.

Apart from physical activity, cognitive activity appears to improve cognition like switching between tasks, visual search or working memory in older adults, as also clearly seen in the above-mentioned study [59, 60]; see also [56, 67] for overviews. Since cognitive and physical training appear to tackle different aspects of cognition it should be advantageous to combine both [68]. Indeed, the combination appears to yield larger effects on cognition in older adults than physical or cognitive training alone [69–76]. It has been argued that cognitive and physical training produce synergistic effects than either one individually [56, 77]. However, the underlying biological and functional mechanisms of the synergistic effects are currently unknown. Hence, more combination studies using neuroimaging methods are necessary. Also, apart from combining aerobic and coordinative training, dancing requires multiple cognitive functions which confirms its suitability as combined intervention to improve physical and mental wellbeing in older adults.

## Conclusion

Physical activity certainly enhances physical fitness but also cognitive fitness. Physical activity is related to unspecific and specific brain changes, the latter depending on the type of activity. Such brain changes are accompanied by improved cognitive functions. Higher-level functions such as executive functions are more improved than lower-level functions. Combined programs which embrace both aerobic, force and coordination training are more favorable since the different aspects of such training induce different brain and behavioral changes. It is probably even more effective to combine complex physical training with cognitive training. Insofar, natural exercises such as regular dancing which affect physical, coordinative and cognitive functions, offer the maximum benefit to preserve and even improve physical and mental fitness in advanced age. In future studies such combined or natural activities should be explored in intervention trials with adequate active control conditions. From a scientific perspective such studies should not only use subjective and behavioral measures but also non-intrusive neurophysiological methods such as electroencephalography.
